# Early risk stratification of sepsis-related liver injury via machine learning: a multicohort study

**DOI:** 10.3389/fmed.2026.1649101

**Published:** 2026-01-27

**Authors:** Xin Chen, Pinwen Zhou, Jiaqi Wang, Li Zhang, Tingbin Xie, Wei Ma, Xinying Wang

**Affiliations:** Department of General Surgery, Nanjing Jinling Hospital, Affiliated Hospital of Medical School, Nanjing University, Nanjing, China

**Keywords:** machine learning, predictive model, random forest, sepsis, sepsis related liver injury

## Abstract

**Background:**

Sepsis-related liver injury (SRLI) is associated with poor prognosis and high morbidity in septic patients. Early mitigation of liver injury is crucial for improving outcomes in the critically ill. However, early detection and intervention remain challenging, due in part to the lack of effective diagnostic and screening strategies. This study aimed to apply machine learning (ML) approaches to identify significant predictors for the onset of SRLI, with the goal of facilitating early identification of high-risk patients.

**Methods:**

This retrospective study utilized data from the Medical Information Mart for Intensive Care IV (MIMIC-IV) database, divided into training and internal validation cohorts. An additional external validation cohort consisted of 120 sepsis patients from Nanjing Jinling Hospital. We constructed seven ML models and two conventional assessment scales to predict the risk of SRLI in patients who did not meet the SRLI criteria within the first 24 h of ICU admission. The Boruta algorithm was employed for feature selection. Hyperparameter tuning was performed on the training set using grid search. Model performance was evaluated by the area under the receiver operating characteristic curve (ROC-AUC) and precision–recall area under the curve (PR-AUC), along with specificity, sensitivity, accuracy, F1 score. The clinical utility of the models was evaluated using decision curve analysis. Shapley additive explanation (SHAP) was used to provide clinicians with an intuitive understanding of the machine learning model.

**Results:**

After applying exclusion criteria, 9,434 sepsis patients from MIMIC-IV were included for model development. The Random Forest (RF) model demonstrated superior overall predictive performance in internal validation, achieving an area under the curve of 0.867, precision–recall area under the curve of 0.392. Decision curve analysis indicated the RF model provided a positive net benefit across a wide range of high risk thresholds. In the RF model, total bilirubin, international normalized ratio, sequential organ failure assessment, logistic organ dysfunction system, and prothrombin time were the most important indexes during the initial 24 hours following ICU admission, according to SHAP value. In the external validation, the RF model also outperformed all others (ROC-AUC: 0.862, PR-AUC: 0.735).

**Conclusion:**

Our study explored ML-based models for predicting SRLI among sepsis at an earlier stage and the performance of random forest model ranked best. The significant predictive contribution of prothrombin time highlights its potential as a key monitoring marker for early risk stratification in septic patients.

## Introduction

Sepsis is a prevalent and life-threatening condition characterized by organ dysfunction caused by a dysregulated host response to infection (1). As a central organ in the defense against sepsis, the liver plays a critical role in eliminating bacteria and microbiota-derived metabolites, coordinating metabolic adaptation to inflammation, and releasing acute-phase proteins and cytokines (2). However, the liver is highly susceptible to be attacked at any stage during sepsis (3), due to its exposure to microorganisms, endotoxins and metabolites from the gastrointestinal tract via the portal vein or systemic circulation (4). According to current reports, the incidence of SRLI is 11–34.7% (5, 6). Despite SRLI not being the most prevalent complication of sepsis, it has been consistently associated with poor prognosis and increased mortality (7, 8). A significant number of SRLI patients do not receive timely and effective therapy, primarily due to the absence of early diagnostic tools, which often leads to progressive liver failure and multiple organ dysfunction syndrome (9–11). Early identification of high-risk patients, though challenging, is essential for improving sepsis outcomes (12).

Liver dysfunction has been identified as one of initial event in the progression of sepsis (13). Although international normalized ratio (INR) and total bilirubin are utilized to diagnose SRLI, they have limited efficacy in identifying high-risk patients. Some studies have indicated that the baseline levels of serum ApoA5, plasminogen activator inhibitor-1, suppressive monocytes, and other soluble factors could help predict the risk of SRLI (14–16). However, there are numerous challenges in clinical application, due to the high cost of laboratory tests, time-consuming procedures, and uncertain predictive value, thus remaining in the experimental stage. Consequently, there is an urgent need to develop predictors with higher sensitivity, specificity, and predictive value that can be feasibly implemented in clinical practice. Xie et al. identified some independent predictors of SRLI using multivariate logistic regression (LR) based on traditional statistical methods in a small sample. Their finding showed a relatively better accuracy, with an AUC value of 0.832 (6). Nevertheless, LR, a conventional supervised learning algorithm, assumes a linear correlation between the dependent and independent variables. This assumption may oversimplify complex non-linear relationships, thereby reducing the predictive accuracy of the model (17, 18). Therefore, exploring a more effective predictive model is crucial for guiding the treatment of sepsis.

Different emerging machine learning (ML) algorithms have been increasingly applied to develop prediction models for sepsis and its related complications in the last few years. Compared to the classic critical care scoring system, such as sequential organ failure assessment (SOFA), acute physiology and chronic health evaluation II (APACHE II), simplified acute physiology score II(SAPS II), ML has demonstrated superior predictive performance (19–21). Collectively, these studies indicate that ML models can effectively predict ICU-related outcomes and enhance the predictive utility of models in intensive care research. Furthermore, ML has been successfully implemented in various liver diseases, including drug-induced liver injury, immune-related hepatic injury, and non-alcoholic fatty liver disease–related fibrosis and cirrhosis, various studies that employ machine learning are gradually verified (22–24).

Yet, there is still lack of evidence supporting the effectiveness of ML in predicting SRLI in sepsis. Thus, we aim to develop, calibrate, and validate the most valuable ML model for predicting SRLI. By leveraging routinely available clinical data, we hope to enable more critically ill patients to benefit from data-driven risk stratification and ultimately reduce the healthcare burden. We incorporated demographic characteristics, laboratory parameters, and procedural information collected within the first 24 hours after ICU admission. Subsequently, seven ML models and two conventional scoring systems were developed to predict the occurrence of SRLI throughout the entire ICU stay. Additionally, Shapley additive explanation (SHAP) was applied to enhance interpretability and provide a better insight into the performance of prediction models.

## Materials and methods

### Data source

The data utilized in this retrospective study were sourced from the Medical Information Mart for Intensive Care IV database (MIMIC-IV) version 2.2 (25), a comprehensive critical care medicine database that includes information on patients admitted to Beth Israel Deaconess Medical Center (BIDMC) from 2008 to 2019. The database comprises admission data, vital sign measurements, laboratory test results, intravenous administrations, ventilator settings, imaging reports, and discharge or death records, generated by the computational physiology laboratory at Massachusetts Institute of Technology (MIT) and endorsed by the institutional review boards of both MIT and BIDMC. All protected health information was deidentified on this platform, which negated the requirement for ethical approval and patient consent for this study (26, 27). One of the authors (Xin Chen) was granted authorization to access data from the database for research endeavors after completion of the Protecting Human Research Participants training course provided by the National Institutes of Health (NIH) website (Certification No. 56135068).

### Patients and SRLI

The study cohort was selected from the MIMIC database according to the following criteria: (1) initial admission to the ICU; (2) individuals aged 18 or older; (3) ICU admission lasting more than 48 hours; (4) satisfying the diagnostic criteria for Sepsis 3.0. The patients mentioned above constituted the training and internal validation cohort.

**FIGURE 1:**
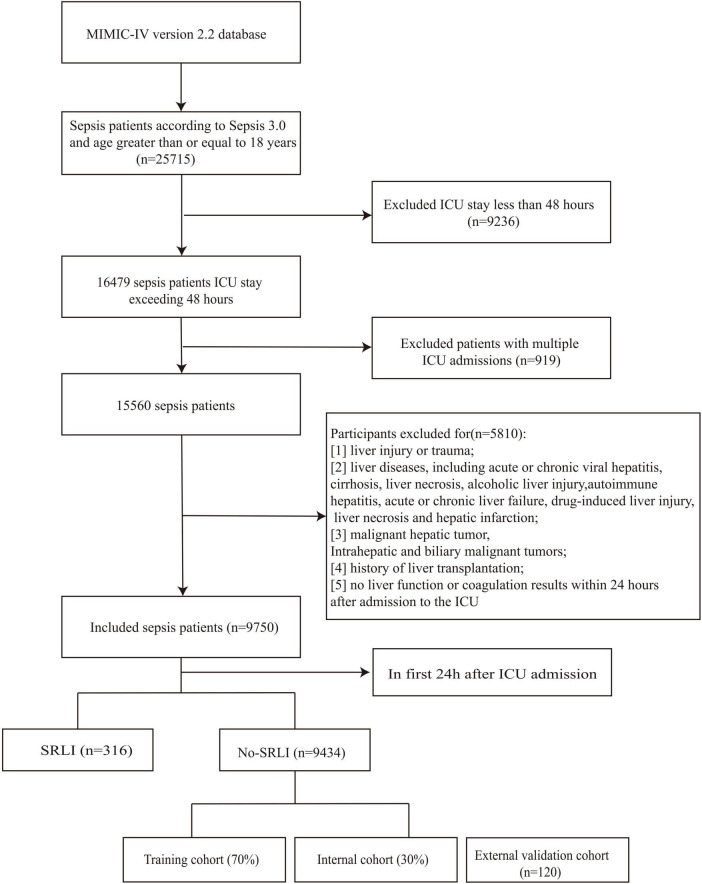
Flowchart of screening sepsis patients.

Participants were excluded if they met any of the following criteria: (1) liver injury or trauma; (2) liver diseases, including acute or chronic viral hepatitis, cirrhosis, liver necrosis, alcoholic liver injury, autoimmune hepatitis, acute or chronic liver failure, drug-induced liver injury, liver necrosis and hepatic infarction; (3) malignant hepatic tumor, Intrahepatic and biliary malignant tumors; (4) history of liver transplantation; (5) no liver function or coagulation results within 24 h after admission to the ICU. Patients who already met SRLI diagnostic criteria within the first 24 h after ICU admission were excluded from both the training and validation cohorts to prevent potential label leakage and ensure model robustness (Figure 1). Patients treated at Nanjing Jinling Hospital (Jiangsu, China) from 2020 to 2023 were recruited for external validation of the model. The inclusion and exclusion criteria were identical to those applied in the training cohort. This research received approval from the Ethics Committee of Nanjing Jinling Hospital (Ethics number: 2024DZKY-002-01).

Based on the Surviving Sepsis Campaign (SSC) guidelines, sepsis-related liver injury is diagnosed when total bilirubin exceeds 34.2 μmol/L (2 mg/dL) along with INR > 1.5 (3, 28). The time-lag prediction window was defined as the first 24 h following ICU admission. The outcome was defined as the development of SRLI occurring after the first 24 h of ICU stay.

### Data extraction and preprocessing

Initially, we used Navicat Premium (version 15.0.12) and PostgreSQL software (version 11.2) to acquire raw data, encompassing sociodemographic characteristics, laboratory parameters, and vital signs (29). The following information was retrieved: (1) Demographic characteristics, including ethnic identity, age, and sex; (2) Comorbid conditions, such as diabetes, hypertension, heart failure, chronic lung disease, and hypertension; (3) Vital signs, such as oxygen saturation (SpO_2_), diastolic blood pressure (DiasBP), systolic blood pressure (SysBP), and heart rate; (4) laboratory indicators, including total bilirubin, INR, prothrombin time (PT), chloride, sodium, potassium, partial thromboplastin time (PTT), albumin, creatinine, glucose, blood urea nitrogen (BUN), and lactate; (5) clinical interventions, encompassing mechanical ventilation, renal replacement therapy, and the utilization of vasopressor. In the analysis, we used the maximum and minimum values for some variables that had multiple measurements. SAPS II and SOFA scores were evaluated based on their initial test values. Comorbidities were identified using the Charlson index. The epidemiological nature of this study meant that no sample size estimation was conducted. To achieve the greatest statistical power, all eligible patients from the MIMIC-IV database were considered.

### Missing data handling

**FIGURE 2:**
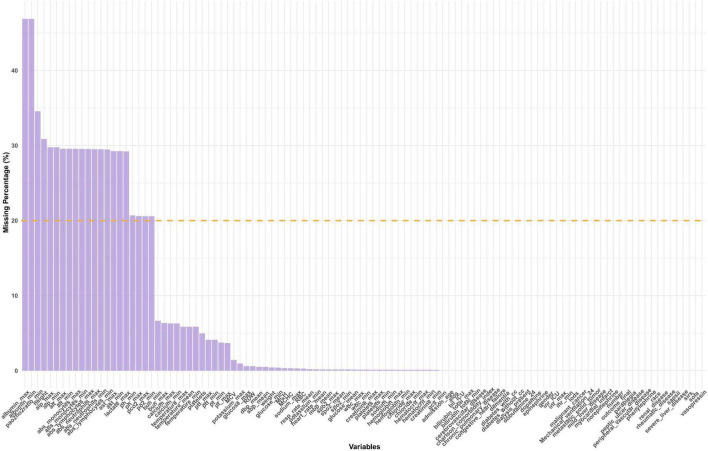
Distribution of missing data. SAPS II, simplified acute physiology score II; GCS, glasgow coma score; SIRS, systemic inflammatory response syndrome; SOFA, sequential organ failure assessment; LODS, logistic organ dysfunction system; SpO2, oxyhemoglobin saturation; Meanbp, mean arterial pressure; SysBP, systolic blood pressure; DiasBP, diastolic blood pressure; BUN, blood urea nitrogen; INR, international normalized ratio; PT, prothrombin time; PTT, partial thromboplastin time; MCH, mean corpuscular hemoglobin; WBC, white blood cell; RBC, red blood cell; RDW, red blood cell distribution width; MCHC, mean corpuscular hemoglobin concentration; MCV, mean corpuscular volume.

For each variable, the proportion of missing values was calculated and visualized (Figure 2). Variables with more than 20% missing data, such as CK-MB, fibrinogen, and absolute lymphocyte count, were excluded from further analysis to reduce potential bias and ensure the robustness of downstream modeling.

Missing values in the retained variables were imputed using multiple imputation by chained equations (MICE) (30). Variable-specific imputation strategies were applied according to data type. Continuous variables were imputed using predictive mean matching (PMM), preserving the original data distribution and avoiding implausible values. Five imputed datasets were generated with 50 iterations each to ensure stable convergence. One completed dataset was used for subsequent modeling.

To evaluate the adequacy of the imputation process, density plots (Supplementary Figure 1) were visually assessed as a distributional consistency between observed and imputed values. Additionally, mean and standard deviation before and after imputation were compared for all numeric variables, confirming minimal distributional distortion (Supplementary Table 1). A quasi–Gelman–Rubin diagnostic based on chain means was further calculated for each variable (Supplementary Table 2).

### Feature selection

To reduce dimensionality and identify the most relevant predictors, feature selection was performed using the Boruta algorithm. Boruta iteratively compared the importance of original features with that of shadow features generated by permutation, classifying predictors as confirmed, tentative, or rejected (31). Only features confirmed as confirmed by Boruta were retained for subsequent model development.

### Model development and evaluation

**FIGURE 3:**
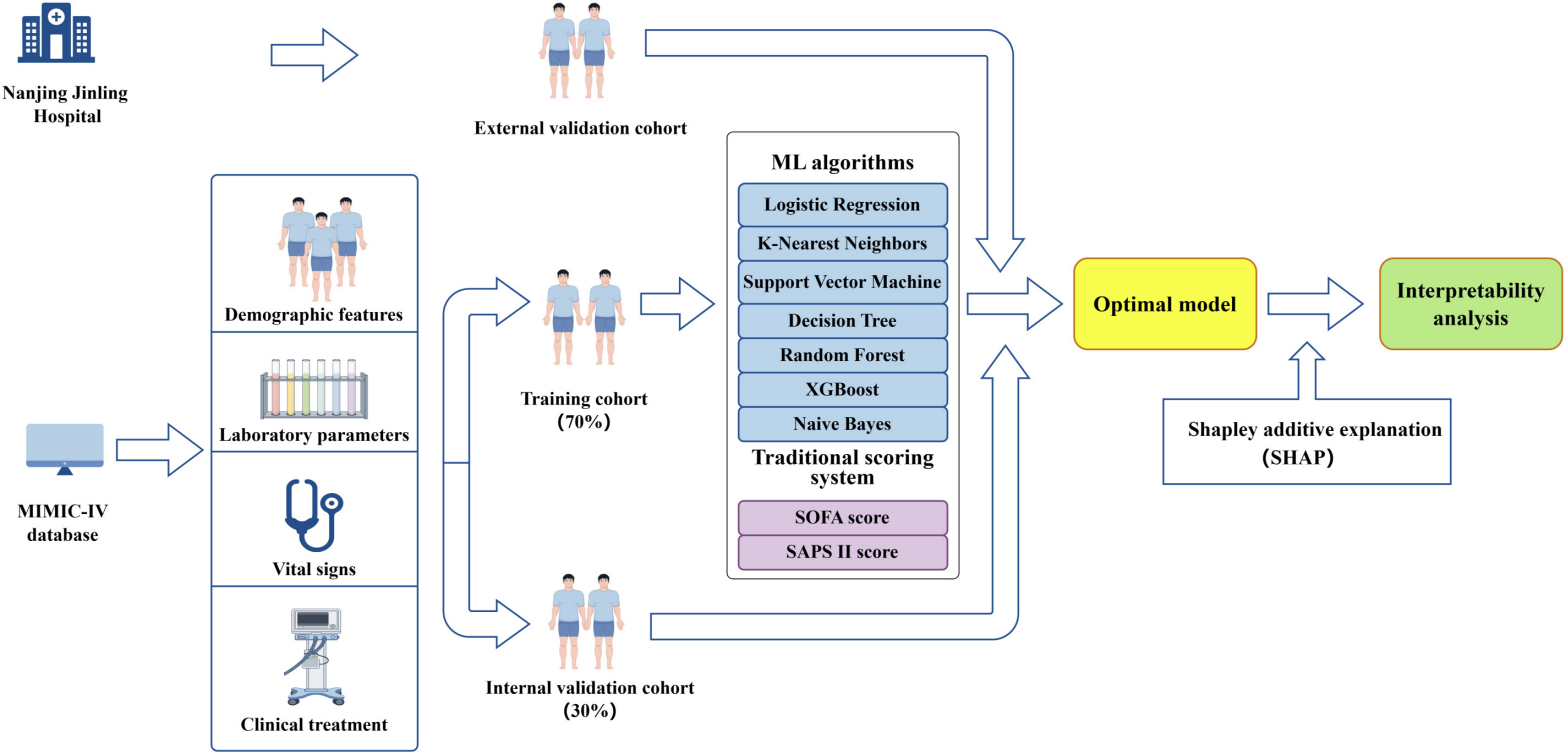
Diagram illustrating the study design (by Figdraw).

The complete dataset was randomly divided into training set and internal validation set using a stratified sampling strategy. Specifically, 70% of the data were allocated to the training set and the remaining 30% to the internal validation set (Figure 3). Stratification was performed based on the outcome variable to ensure that the proportion of positive and negative cases was preserved across both subsets. To address class imbalance in the training data, the Synthetic Minority Over-sampling Technique (SMOTE) was applied (32). Multiple machine learning models were developed, including random forest (RF), support vector machine (SVM) with radial basis kernel, k-nearest neighbors (KNN), extreme gradient boosting (XGBoost), decision tree (DT), naïve Bayes (NB), and logistic regression (LR). Hyperparameter tuning was performed on the training dataset using grid search within parameter ranges informed by prior literature, and model performance was subsequently assessed on the internal validation dataset. For the random forest model, the number of trees, the number of variables randomly sampled at each split, and the minimum node size were tuned. For the support vector machine with radial basis function kernel, the regularization parameter and kernel width were optimized. The k-nearest neighbors’ model was optimized across a range of neighborhood sizes, with feature scaling applied prior to model fitting. Hyperparameters include the number of boosting iterations, learning rate, maximum tree depth, minimum loss reduction, subsampling ratio, and column sampling ratio were tuned for the XGBoost model. Decision tree model was optimized by varying the complexity parameter, maximum tree depth, and minimum node size, while naïve Bayes models were tuned by adjusting kernel density estimation and Laplace smoothing parameters. Logistic regression was fitted without additional hyperparameter tuning and served as a baseline model.

Hyperparameter selection was guided by maximizing the F1 score on the internal validation set. The F1 score was chosen as the primary optimization metric to balance precision and recall. After identifying the optimal hyperparameter configuration for each model, the final model was refitted on the full training data using the selected parameters and subsequently evaluated on the internal validation set for performance comparison. The optimal classification threshold for each model was determined by maximizing the F1 score.

Model performance was assessed using receiver operating characteristic area under the curve (ROC-AUC), precision–recall area under the curve (PR-AUC), accuracy, recall, sensitivity, specificity, F1 score, and Brier score. Calibration was further evaluated using calibration slope and intercept derived from logistic regression of observed outcomes on predicted probabilities. In addition, ROC curves were generated to facilitate visual comparison of discriminative performance among the different models

### Decision curve analysis and model calibration

To evaluate the potential clinical utility of the predictive models across a range of decision thresholds, decision curve analysis (DCA) was performed. Based on predicted probabilities in the validation cohort, net benefit was calculated over a clinically relevant range of threshold probabilities and compared with default strategies of treating all patients or treating none.

Given that tree-based ensemble models may exhibit suboptimal probability calibration, the random forest model was further subjected to probability calibration. Isotonic regression was applied to predict probabilities in the validation set to improve agreement between predicted risks and observed outcome frequencies. Model performance before and after calibration was evaluated separately. Calibration curves were constructed by comparing predicted probabilities with observed event rates across probability strata, allowing visual assessment of calibration performance and the impact of isotonic calibration.

### Visualization of random forest feature importance

To explore the relative contribution of individual predictors within the random forest model, feature importance was quantified using the model’s internal importance measures. Features were ranked according to their contribution to reducing classification error or node impurity. The top ten most important variables were selected and visualized in a feature importance plot, providing an overview of the key predictors driving the model’s discriminative performance and highlighting clinically relevant variables.

### SHAP-based global model interpretation

To enhance model interpretability beyond traditional feature importance metrics, Shapley Additive Explanations (SHAP) were employed to interpret the random forest model. SHAP assigns each feature an importance value for individual predictions by quantifying the marginal contribution relative to all possible feature combinations. SHAP values were calculated for each sample in the validation set, resulting in a SHAP value matrix used for further interpretation.

Global model interpretation was performed by ranking features according to the mean absolute SHAP value, identifying variables with the greatest overall impact on model predictions. Subsequently, SHAP global importance plot was constructed to visualize both the magnitude and direction of feature effects, illustrating how varying feature values contribute to higher or lower predicted risk.

### SHAP dependence plots and identification of risk inflection points

For the most influential continuous variables identified by SHAP analysis, SHAP dependence plots were generated to visualize the relationship between feature values and their corresponding SHAP values. These plots characterize potential non-linear and threshold effects of predictors on model output. To identify clinically relevant risk thresholds, we analyzed the smoothed SHAP, defined as crossing points where SHAP values intersected zero (SHAP = 0). These points were interpreted as potential risk thresholds, indicating values beyond which a predictor exerts a qualitatively different or stronger effect on outcome risk, thereby offering clinically interpretable cutoff insights.

### Individual-level explanation using SHAP force plots

To provide individualized explanations of model predictions, SHAP force plots were constructed for two representative patients from the internal validation cohort, one with a negative outcome and one with a positive outcome. These force plots illustrate how individual features contribute either positively or negatively to the predicted risk for each patient, and how the combined effects of all variables drive the model output toward a higher or lower probability of SRLI.

## Results

### Baseline characteristics

Sepsis was confirmed in 25,715 patients during initial hospitalization. A total of 15,965 patients were excluded based on the predefined exclusion criteria (Figure 1). Ultimately, 9750 patients met the inclusion criteria, among whom 316 patients (3.2%) developed sepsis-related liver injury (SRLI) within the first 24 h after ICU admission. After excluding these patients, we randomly assigned the remaining patients (*n* = 9,434) into the training and validation cohorts at a ratio of 7:3. Comparison of clinical characteristics between SRLI (*n* = 350) and non-SRLI (*n* = 6,255) groups of the training cohort are detailed in Table 1. Male patients showed a higher predisposition to SRLI during hospitalization. SRLI patients exhibited higher body weight and a greater prevalence of congestive heart failure, peripheral vascular disease, and malignancy cancer, while lower incidences of cerebrovascular disease, and paraplegia were observed. SRLI patients also showed increased usage of dialysis and mechanical ventilation compared to those without SRLI. Additionally, SRLI patients demonstrated significantly greater illness severity (SOFA score, SAPS II score, LODS score, and SIRS score) and higher levels of prothrombin time (PT), partial thromboplastin time (PTT), glucose and creatinine than non-SRLI patients. compared to non-SRLI sepsis patients (*P* < 0.05). However, hemoglobin, platelet, red blood cell, SysBP, DiasBP, and MeanBP were lower in SRLI group than in non-SRLI group (*P* < 0.05).

**TABLE 1:** Baseline characteristics of septic patients in the training cohort.

Variables	Total (*n* = 6,605)	Non-SRLI (*n* = 6,255)	SRLI (*n* = 350)	*P-*value
Gender				0.044
Female (%)	2,949 (44.65)	2,811 (44.94)	138 (39.43)	
Male (%)	3,656 (55.35)	3,444 (55.06)	212 (60.57)	
Age (year)	70.34 (58.80, 80.40)	70.33 (58.49, 80.41)	70.86 (61.00, 80.21)	0.571
Weight (kg)	78.80 (65.80, 94.80)	78.60 (65.67, 94.50)	82.00 (67.60, 97.94)	0.003
SOFA	6.00 (4.00, 9.00)	6.00 (4.00, 9.00)	10.00 (7.00, 12.00)	< 0.001
SAPSII	40.00 (32.00, 49.00)	40.00 (32.00, 49.00)	48.00 (38.00, 55.00)	< 0.001
GCS	13.00 (8.00, 14.00)	13.00 (8.00, 14.00)	11.00 (6.00, 14.00)	< 0.001
LODS	6.00 (4.00, 9.00)	6.00 (4.00, 8.00)	9.00 (6.00, 11.00)	< 0.001
SIRS	3.00 (2.00, 3.00)	3.00 (2.00, 3.00)	3.00 (3.00, 4.00)	< 0.001
**Comorbidity**				
Myocardial infarct, n (%)	1,394 (21.11)	1,304 (20.85)	90 (25.71)	0.030
Congestive heart failure, n (%)	2,384 (36.09)	2,224 (35.56)	160 (45.71)	< 0.001
Peripheral vascular disease, n (%)	958 (14.50)	865 (13.83)	93 (26.57)	< 0.001
Cerebrovascular disease, n (%)	1,293 (19.58)	1,261 (20.16)	32 (9.14)	< 0.001
Chronic pulmonary disease, n (%)	1,865 (28.24)	1,759 (28.12)	106 (30.29)	0.381
Renal disease, n (%)	1,647 (24.94)	1,538 (24.59)	109 (31.14)	0.006
Malignant cancer, n (%)	864 (13.08)	800 (12.79)	64 (18.29)	0.003
Metastatic solid tumor, n (%)	436 (6.60)	402 (6.43)	34 (9.71)	0.016
**Vital signs^a^**				
SysBP_mean (mmHg)	113.35 (104.73, 125.11)	113.83 (105.08, 125.62)	107.24 (100.88, 116.26)	< 0.001
DiasBP_mean (mmHg)	60.38 (54.39, 67.58)	60.53 (54.49, 67.88)	57.67 (52.73, 63.32)	< 0.001
Meanbp_mean (mmHg)	75.43 (69.80, 82.60)	75.61 (69.88, 82.83)	72.71 (68.06, 77.67)	< 0.001
Resprate_mean (min^–1^)	19.33 (17.07, 22.26)	19.30 (17.05, 22.21)	19.85 (17.37, 22.83)	0.027
Temperature_min (°C)	36.44 (36.00, 36.72)	36.44 (36.00, 36.72)	36.39 (35.67, 36.61)	< 0.001
Temperature_max (°C)	37.44 (37.06, 38.06)	37.44 (37.06, 38.06)	37.39 (36.94, 38.00)	0.055
Temperature_mean (°C)	36.90 (36.61, 37.28)	36.91 (36.62, 37.29)	36.82 (36.47, 37.19)	< 0.001
SpO2_min (%)	93.00 (90.00, 95.00)	93.00 (90.00, 95.00)	92.00 (89.00, 94.75)	0.002
SpO2_max (%)	100.00 (100.00, 100.00)	100.00 (100.00, 100.00)	100.00 (100.00, 100.00)	0.057
SpO2_mean (%)	97.41 (95.88, 98.73)	97.41 (95.89, 98.74)	97.44 (95.78, 98.54)	0.290
**Laboratory parameters^b^**				
Hematocrit_min (%)	29.50 (25.20, 34.30)	29.60 (25.30, 34.50)	26.60 (23.20, 31.08)	< 0.001
Hematocrit_max (%)	34.60 (30.40, 39.30)	34.60 (30.40, 39.40)	33.60 (29.82, 38.10)	0.008
Hemoglobin_min (g/dL)	9.70 (8.30, 11.30)	9.80 (8.30, 11.40)	8.80 (7.70, 10.30)	< 0.001
Hemoglobin_max (g/dL)	11.30 (9.90, 13.00)	11.30 (9.90, 13.00)	11.10 (9.72, 12.50)	0.038
Platelet_min (10^9^/L)	175.00 (122.00, 242.00)	178.00 (125.00, 244.00)	133.00 (83.25, 190.00)	< 0.001
Platelet_max (10^9^/L)	219.00 (161.00, 296.00)	221.00 (162.00, 298.00)	186.00 (130.25, 257.25)	< 0.001
WBC_min (10^9^/L)	10.10 (7.10, 13.70)	10.10 (7.20, 13.70)	9.80 (6.35, 13.40)	0.056
WBC_max (10^9^/L)	14.20 (10.20, 19.20)	14.20 (10.20, 19.10)	15.30 (10.50, 20.50)	0.065
BUN_min (mg/dl)	19.00 (13.00, 31.00)	19.00 (13.00, 31.00)	24.00 (15.00, 36.00)	< 0.001
BUN_max (mg/dL)	24.00 (16.00, 38.00)	23.00 (16.00, 38.00)	28.50 (19.00, 44.00)	< 0.001
Chloride_min (mEq/L)	102.00 (98.00, 106.00)	102.00 (98.00, 106.00)	102.00 (98.00, 106.00)	0.360
Chloride_max (mEq/L)	107.00 (103.00, 110.00)	107.00 (103.00, 110.00)	106.00 (102.00, 111.00)	0.658
Creatinine_min (mg/dL)	0.90 (0.70, 1.40)	0.90 (0.70, 1.40)	1.10 (0.80, 1.70)	< 0.001
Creatinine_max (mg/dL)	1.20 (0.80, 1.80)	1.20 (0.80, 1.80)	1.40 (1.00, 2.20)	< 0.001
Glucose_min (mg/dL)	114.00 (95.00, 137.00)	114.00 (95.00, 137.00)	111.00 (95.00, 137.00)	0.816
Glucose_max (mg/dL)	151.00 (122.00, 201.00)	151.00 (122.00, 200.00)	159.00 (123.25, 215.50)	0.033
Sodium_min (mEq/L)	137.00 (134.00, 140.00)	137.00 (134.00, 140.00)	136.00 (133.00, 139.00)	< 0.001
Sodium_max (mEq/L)	140.00 (138.00, 143.00)	140.00 (138.00, 143.00)	140.00 (137.00, 142.00)	0.005
Potassium_min (mEq/L)	3.80 (3.50, 4.20)	3.80 (3.50, 4.20)	3.80 (3.50, 4.30)	0.520
Potassium_max (mEq/L)	4.50 (4.10, 5.00)	4.50 (4.10, 5.00)	4.60 (4.20, 5.10)	0.002
INR_min	1.20 (1.10, 1.30)	1.20 (1.10, 1.30)	1.30 (1.20, 1.50)	< 0.001
INR_max	1.30 (1.18, 1.50)	1.30 (1.10, 1.50)	1.60 (1.39, 2.00)	< 0.001
PT_min (s)	13.30 (12.10, 15.00)	13.20 (12.00, 14.90)	14.65 (13.30, 16.87)	< 0.001
PT_max (s)	14.50 (12.90, 17.00)	14.40 (12.80, 16.80)	17.85 (15.13, 21.30)	< 0.001
PTT_min (s)	28.50 (25.60, 32.80)	28.40 (25.50, 32.50)	31.40 (28.10, 36.88)	< 0.001
PTT_max (s)	32.70 (28.20, 44.50)	32.40 (28.00, 43.40)	42.00 (32.65, 65.63)	< 0.001
Bilirubin_min (mg/dL)	0.50 (0.30, 0.80)	0.50 (0.30, 0.80)	0.95 (0.50, 1.60)	< 0.001
Bilirubin_max (mg/dL)	0.70 (0.40, 1.20)	0.63 (0.40, 1.11)	1.22 (0.80, 1.90)	< 0.001
MCH (pg)	30.00 (28.50, 31.40)	30.00 (28.50, 31.40)	30.20 (28.70, 31.50)	0.222
MCHC (g/L)	32.80 (31.70, 33.90)	32.80 (31.70, 33.90)	33.05 (31.90, 34.00)	0.056
MCV (fl)	91.00 (87.00, 95.00)	91.00 (87.00, 95.00)	91.00 (87.00, 95.00)	0.939
RBC (10^12^/L)	3.59 (3.07, 4.13)	3.60 (3.08, 4.13)	3.46 (2.97, 3.99)	< 0.001
RDW (%)	14.60 (13.60, 16.00)	14.60 (13.50, 16.00)	15.30 (14.00, 16.90)	< 0.001
**Advanced life support**				
Renal replacement therapy (%)	303 (4.59)	276 (4.41)	27 (7.71)	0.004
Mechanical ventilation (%)	3,756 (56.87)	3,534 (56.50)	222 (63.43)	0.011

^a^Vital signs were assessed as minimum, and maximum, and mean values for the first 24 h after ICU admission.

^b^The laboratory parameters documented the lowest and highest values within the first 24 h following ICU admission. SAPS II, simplified acute physiology score II; GCS, glasgow coma score; SIRS, systemic inflammatory response syndrome; SOFA, sequential organ failure assessment; LODS, logistic organ dysfunction system; SpO_2_, oxyhemoglobin saturation; Meanbp, mean arterial pressure; SysBP, systolic blood pressure; DiasBP, diastolic blood pressure; BUN, blood urea nitrogen; INR, international normalized ratio; PT, prothrombin time; PTT, partial thromboplastin time; MCH, mean corpuscular hemoglobin; WBC, white blood cell; RBC, red blood cell; RDW, red blood cell distribution width; MCHC, mean corpuscular hemoglobin concentration; MCV, mean corpuscular volume.

### Features selection

**FIGURE 4:**
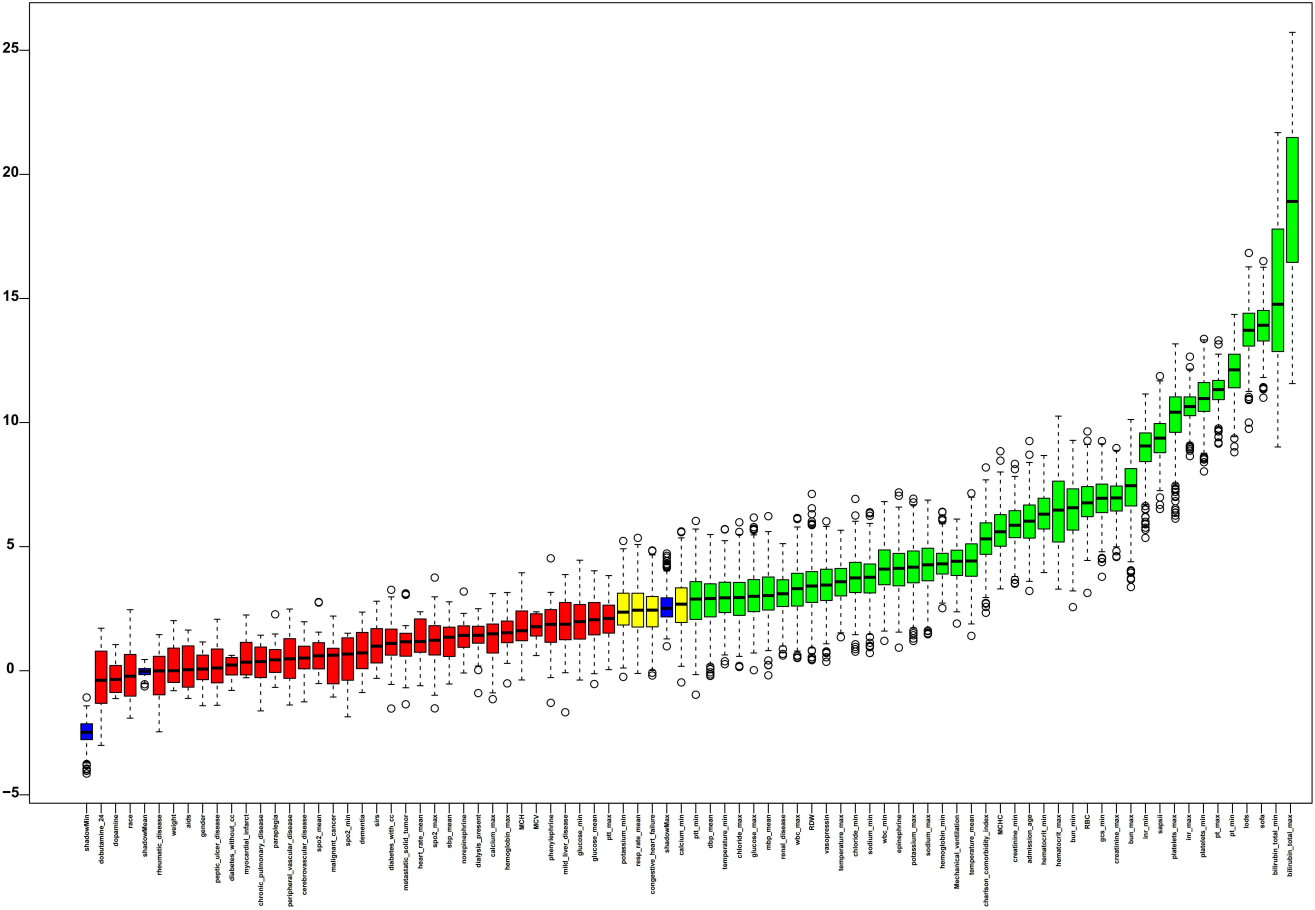
The process of feature selection utilized the Boruta algorithm. Variable names are arranged along the horizontal axis, with their Z-values depicted on the vertical axis. The green boxes highlight the crucial variables, yellow boxes indicate tentative attributes, and red represent unimportant ones. SIRS, systemic inflammatory response syndrome; GCS, glasgow coma score; SAPSII, simplified acute physiology score II; SOFA, sequential organ failure assessment; LODS, logistic organ dysfunction system; SysBP, systolic blood pressure; Meanbp mean arterial pressure; DiasBP, diastolic blood pressure; SpO_2_, oxyhemoglobin saturation; INR, international normalized ratio; WBC, white blood cell; PT, prothrombin time; PTT, partial thromboplastin time; RBC, red blood cell; RDW, red blood cell distribution width; MCH, mean corpuscular hemoglobin; MCV, mean corpuscular volume; MCHC, mean corpuscular hemoglobin concentration.

Figure 4 presented the results of feature selection performed using the Boruta algorithm. The 42 variables shown in green represent higher Z-value and most strongly associated with SRLI occurrence. Key positively contributing variables included: bilirubin_total_max, bilirubin_total_min, sofa, lods, pt_min, pt_max, platelets_min, inr_max, platelets_max, SAPS II.

### Model development and internal validation performance

**FIGURE 5:**
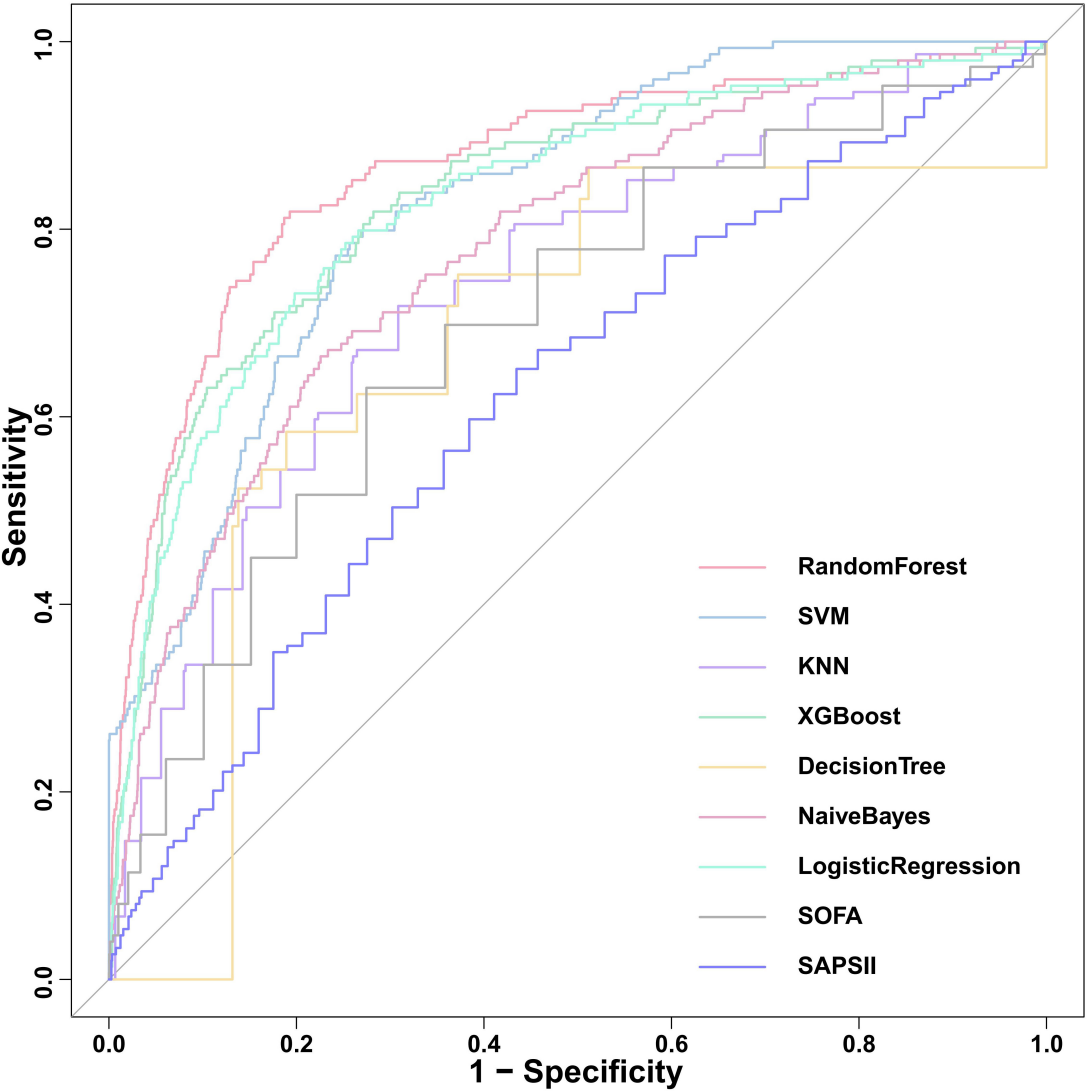
Comparison of ROC curves for the nine predictive models in internal validation cohort. SOFA, sequential organ failure assessment; SAPS II, simplified acute physiology score; XGBoost, Extreme Gradient Boosting; KNN, k-nearest neighbors; SVM, support vector machine.

We constructed seven machine learning models (RF, SVM, KNN, XGB, DT, NB, and LR) and two traditional scoring systems (SOFA and SAPSII) to forecast the onset of SRLI in sepsis after ICU admission. Performance metrics of all models in the internal validation cohort are presented in Table 2. Among all models, the RF model demonstrated the best overall predictive performance, achieving the highest ROC-AUC (0.867, 95% CI: 0.834–0.900), PR-AUC (0.392, 95% CI: 0.308–0.473), along with strong discriminative ability (accuracy: 0.933, specificity: 0.959) compared with other machine learning models. These results indicate that RF not only discriminated well between outcome classes but also provided a favorable trade-off between precision and recall relative to other ML models. Notably, the XGBoost (ROC-AUC: 0.837, 95% CI: 0.802–0.873, PR-AUC: 0.280, 95% CI: 0.216–0.350) and LR (ROC-AUC: 0.830, 95% CI: 0.794–0.866, PR-AUC: 0.276, 95% CI: 0.206–0.346) models also showed better performance. In contrast, the two conventional scoring systems, SOFA (ROC-AUC: 0.729, 95% CI: 0.687–0.771, PR-AUC: 0.167, 95% CI: 0.117–0.225) and SAPSII (ROC-AUC: 0.632, 95% CI: 0.586–0.678, PR-AUC: 0.090, 95% CI: 0.067–0.118) demonstrated substantially lower discriminative performance. The ROC curves for all models were presented in Figure 5.

**TABLE 2:** Summary of performance metrics for the nine predictive models.

Model	ROC-AUC	95% CI		PR-AUC	95% CI		Recall	Accuracy	F1	Sensitivity	Specificity
		Lower	Upper		Lower	Upper					
RF	0.867	0.834	0.900	0.392	0.308	0.473	0.470	0.933	0.424	0.470	0.959
SVM	0.829	0.798	0.859	0.391	0.310	0.461	0.262	0.960	0.408	0.262	0.999
KNN	0.755	0.713	0.796	0.176	0.128	0.233	0.289	0.910	0.252	0.289	0.944
XGBoost	0.837	0.802	0.873	0.280	0.216	0.350	0.530	0.915	0.396	0.530	0.936
DT	0.748	0.708	0.788	0.136	0.107	0.167	0.523	0.844	0.261	0.523	0.862
Naïve Bayes	0.777	0.738	0.816	0.207	0.151	0.270	0.376	0.905	0.294	0.376	0.934
LR	0.830	0.794	0.866	0.276	0.206	0.346	0.450	0.919	0.368	0.450	0.945
SOFA	0.729	0.687	0.771	0.167	0.117	0.225	0.450	0.828	0.215	0.450	0.849
SAPS II	0.632	0.586	0.678	0.090	0.067	0.118	0.349	0.800	0.155	0.349	0.825

RF, random forest; LR, logistic regression; KNN, k-nearest neighbors; XGBoost, Extreme Gradient Boosting; SVM, support vector machine; DT, decision tree; SOFA, sequential organ failure assessment; SAPS II, simplified acute physiology score.

### Calibration performance and decision curve analysis

Calibration performance of the ML models were assessed using calibration curves, calibration slope, intercept, and Brier score (Supplementary Table 3). Given the known tendency of tree-based models to produce poorly calibrated probabilities, we applied isotonic regression to calibrate the output of RF model. Performance before and after calibration is detailed in Supplementary Table 4. After isotonic regression, calibration performance improved substantially. The calibration curve visually aligned closer to the ideal diagonal (Supplementary Figure 2).

**FIGURE 6:**
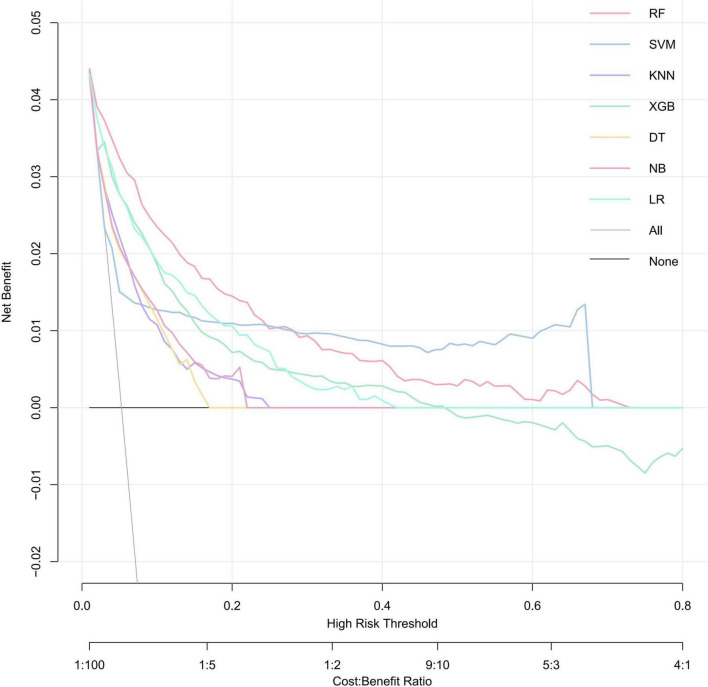
Decision curves for evaluating the clinical utility of ML models in predicting SRLI. A model is considered clinically useful at thresholds where its curve lies above grey solid lines and black lines. The RF model (red curve) demonstrates the widest range of high net benefit. RF, random forest; SVM, support vector machine; KNN, k-nearest neighbors; XGBoost, extreme gradient boosting; DT, decision tree; NB, naïve Bayes; LR, logistic regression.

DCA was performed to evaluate the net clinical benefit of each prediction model (Figure 6). RF model provided the highest net benefit across the widest range of threshold probabilities among all compared models. The net benefit of the RF exceeded that of treat-all and treat-none strategies for threshold probabilities ranging from approximately 2–72%. This wide interval encompasses most clinically plausible decision thresholds. Within the threshold range of approximately 2–25%, its net benefit surpassed that of all other comparative models, suggesting the potential utility of RF model in guiding clinical decisions when intervention thresholds fall within this interval.

### Model interpretability analysis

**FIGURE 7:**
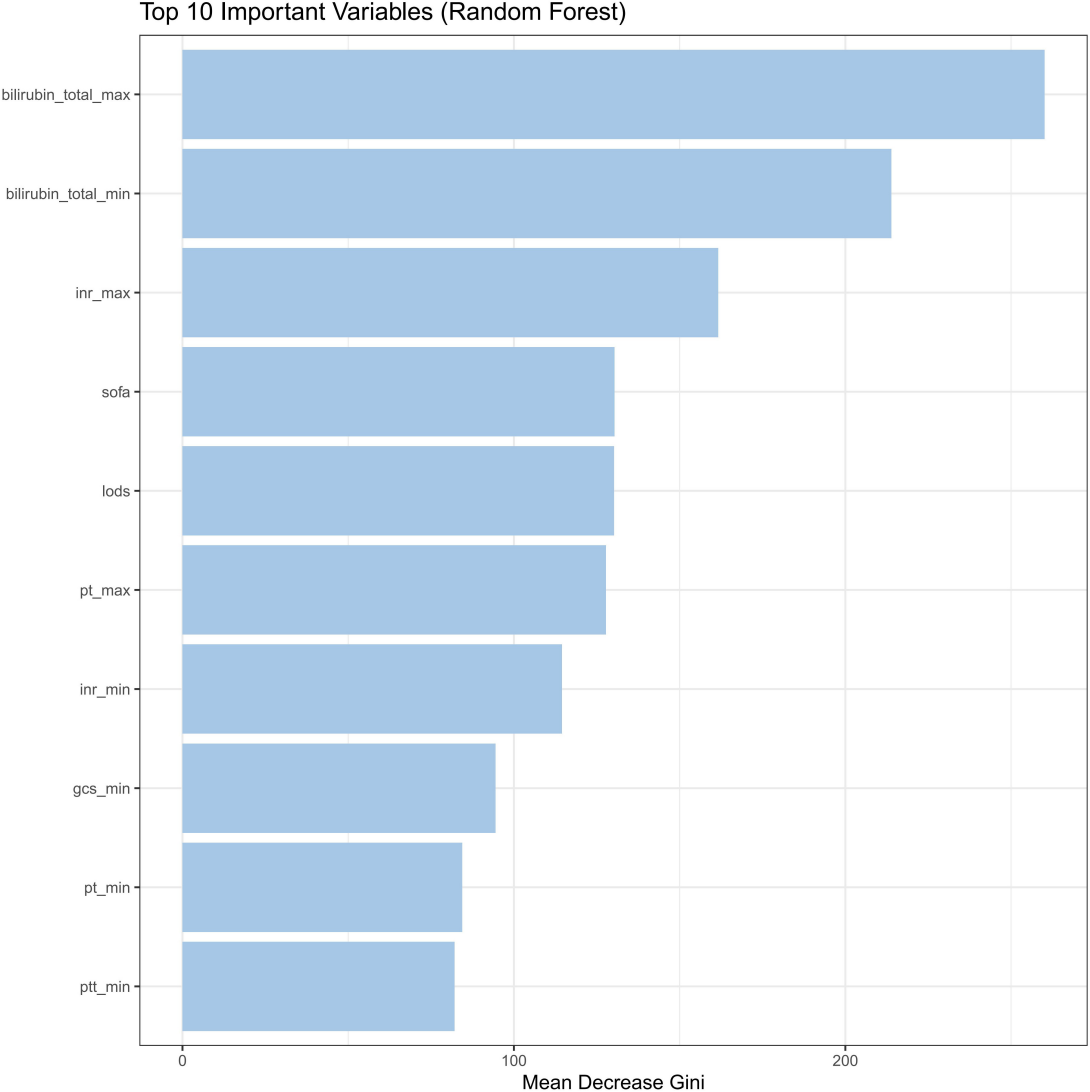
Feature importance by the mean decrease Gini. INR, international normalized ratio; SOFA, sequential organ failure assessment; LODS, logistic organ dysfunction system; PT, prothrombin time; GCS, glasgow coma score; PTT, partial thromboplastin time.

**FIGURE 8:**
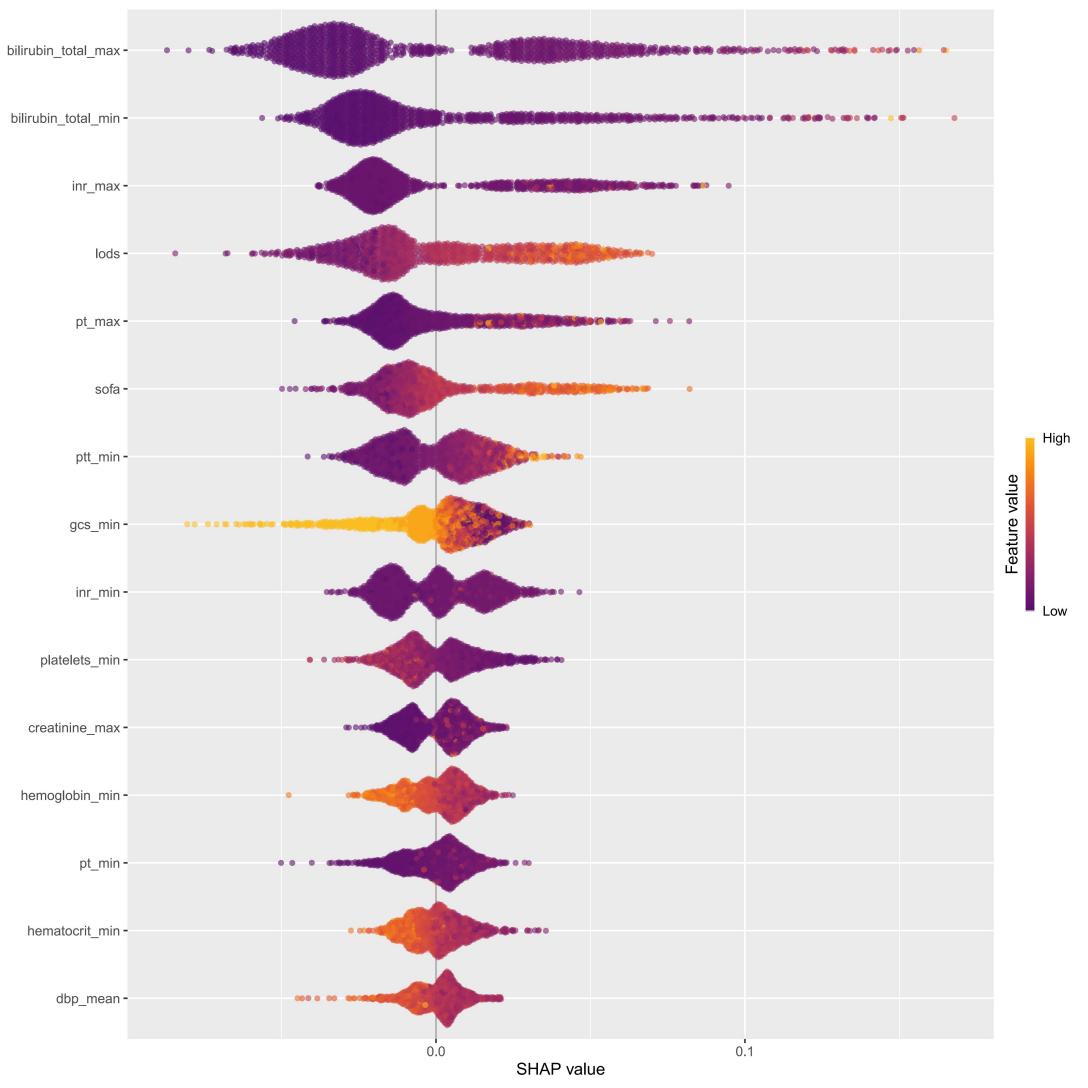
Feature importance was evaluated for the RF model using SHAP analysis. The SHAP summary plot illustrated the 15 most influential features, with each dot representing the SHAP value for a single patient. The color of each dot reflected the corresponding feature value (yellow denotes a high value, while purple denotes a low value), and the density of points along each row showed their distribution. Furthermore, points that were farther from the SHAP value of baseline had a more significant effect on the output. INR, international normalized ratio; LODS, logistic organ dysfunction system; PT, prothrombin time; SOFA, sequential organ failure assessment; PTT, partial thromboplastin time; GCS, glasgow coma score; dbp, diastolic blood pressure.

The intrinsic feature importance of the RF model, quantified by the mean decrease Gini, identified the ten most influential predictors for discriminating SRLI risk. As shown in Figure 7, total bilirubinn emerged as the most important predictor, followed by INR, SOFA score, and the LODS score. Other notable variables included PT and GCS. To further elucidate the superior performance of the RF model, we comprehensively investigated the global and local interpretability. The significance of features was evaluated using the entire sample from the training group. We assessed the global importance of each feature using SHAP values to comprehend the impact of various features across all samples. The SHAP summary plot illustrated the 15 variables with the most significant impact on model output which reaffirmed the critical role of bilirubin_total, inr, pt, and lods, consistent with the mean decrease Gini (Figure 8).

SHAP dependence plots were generated for the top-ranked features to explore potential non-linear relationships between individual predictors and the model output (Supplementary Figure 3). Clear threshold effects were observed for the key variables. For bilirubin_total_max, the impact on risk accelerated markedly beyond 0.75 mg/dL, and values exceeding 0.80 mg/dL consistently contributed to increased risk. A similar pattern was observed for the INR, with SHAP values rising markedly when values exceeding 1.45. LODS, SOFA, pt, and ptt also demonstrated threshold-like behaviors, with higher values consistently contributing positively to SRLI risk. Quantitative summaries of these threshold values are presented in Supplementary Table 5.

**FIGURE 9:**
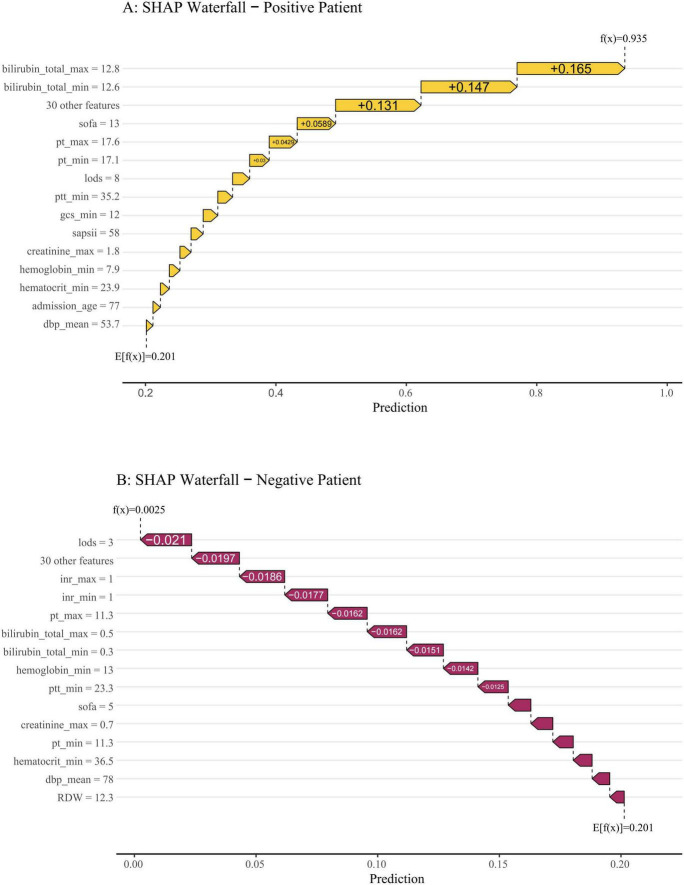
SHAP waterfall plots illustrate individualized predictions for two representative patients. The base value (0.201) represents the average predicted risk. Features are ranked from bottom to top by the magnitude of their SHAP contribution. Feature values are listed on the left, and their respective impacts—pushing the prediction higher (to the right, in yellow) or lower (to the left, in red)—are shown as horizontal arrows. **(A)** A high-risk patient with a final prediction of f(x) = 0.935. The risk was driven upward primarily by markedly elevated total bilirubin (max: 12.8 mg/dL, min: 12.6 mg/dL), a high SOFA score (13), prolonged PT, and a heightened LODS score. **(B)** A low-risk patient with a final prediction of f(x) = 0.0025. The prediction was pulled far below the baseline mainly by favorable values, including a low LODS score (3), normal INR (1.0), and low bilirubin levels.

To visually demonstrate how individual features contribute to patient-specific predictions, we employed SHAP waterfall plots to interpret two representative cases from the validation cohort (Figure 9). This explanation starts from the base value, defined as the average model prediction over the entire dataset. Each feature is then represented by an arrow that pushes the final prediction value higher (to the right) or lower (to the left) from this baseline. The arrow length corresponds to the magnitude of the feature’s contribution, with colors indicating the direction: yellow denotes risk-increasing effects and red indicates risk-decreasing effects. The base value was 0.201. As shown in Figure 9A, the output value for the first case was 0.935, which is higher than the base value, indicating a relatively high predicted probability of SRLI. This elevated risk was primarily driven by high levels of total bilirubin, SOFA score, and PT. In contrast, the second case (Figure 9B) exhibited a significantly lower output value of 0.0025, reflecting a very low predicted risk. This was largely attributed to favorable values of key protective features, such as low LODS, normal INR and PT. These case-specific explanations transparently illustrate how the model integrates multiple, clinical variables to generate a personalized risk assessment, thereby bridging computational predictions with clinical reasoning.

### External validation

**FIGURE 10:**
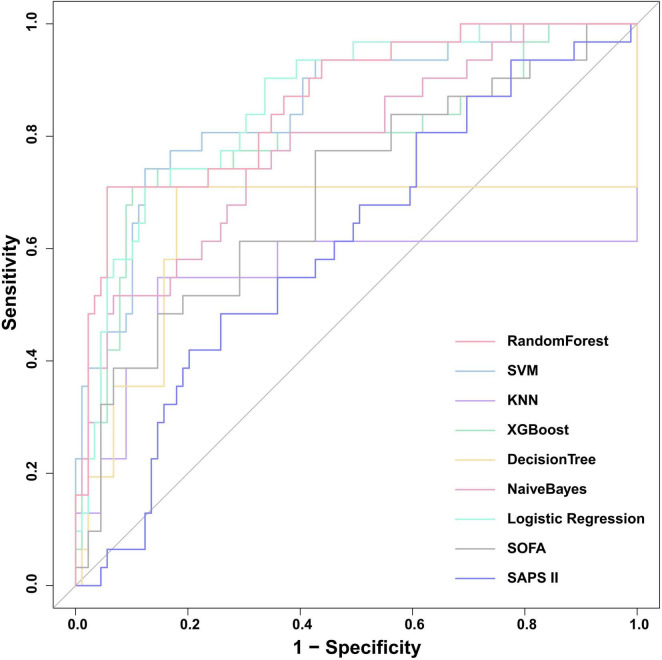
ROC curves for the nine models compared in the external validation cohort. SOFA, sequential organ failure assessment; SAPS II, simplified acute physiology score II; KNN, k-nearest neighbors; XGBoost, extreme gradient boosting; SVM, support vector machine.

We validated the external cohort enrolled from Nanjing Jinling Hospital (Jiangsu, China) between 2021 and 2023. As shown in Figure 10, the RF model achieved the highest discriminative performance with an ROC-AUC of 0.862 (95% CI: 0.785–0.938), and PR-AUC of 0.735 (95% CI: 0.575–0.873), with the highest F1-score (0.759), accuracy (0.883), and balanced sensitivity (0.710) and specificity (0.944) (Table 3). It was followed by Logistic Regression (ROC-AUC: 0.859, 95% CI: 0.786-0.932, PR-AUC: 0.677, 95% CI: 0.503–0.845) and SVM (ROC-AUC: 0.853, 95% CI: 0.773–0.933, PR-AUC: 0.723, 95% CI: 0.542–0.850). Calibration metrics for all models are detailed in Supplementary Table 6. Analysis of the confusion matrices (Supplementary Table 7) revealed that the RF model correctly identified 22 true positives against 5 false negatives, while maintaining 84 true negatives and 9 false positives. This performance profile underscores a high-specificity predictive strategy. Conversely, the SAPSII model showed higher sensitivity (0.806) but substantially lower specificity (0.337), resulting in a markedly higher false-positive rate. Collectively, the external validation confirms that the RF model generalizes effectively to an independent patient population, preserving its superior discriminative power.

**TABLE 3:** Evaluation of the nine models on the external validation cohort.

Model	ROC-AUC	95% CI		PR-AUC	95% CI		Recall	Accuracy	F1	Sensitivity	Specificity
		Lower	Upper		Lower	Upper					
RF	0.862	0.785	0.938	0.735	0.575	0.873	0.710	0.883	0.759	0.710	0.944
SVM	0.853	0.773	0.933	0.723	0.542	0.850	0.645	0.833	0.667	0.645	0.899
KNN	0.688	0.575	0.802	0.543	0.367	0.708	0.548	0.775	0.557	0.548	0.854
XGBoost	0.798	0.694	0.902	0.645	0.432	0.810	0.710	0.850	0.710	0.710	0.899
DT	0.758	0.659	0.856	0.537	0.349	0.726	0.710	0.792	0.638	0.710	0.820
Naïve Bayes	0.780	0.682	0.877	0.627	0.437	0.779	0.516	0.825	0.604	0.516	0.933
LR	0.859	0.786	0.932	0.677	0.503	0.845	0.710	0.825	0.677	0.710	0.865
SOFA	0.736	0.630	0.842	0.532	0.349	0.712	0.774	0.625	0.516	0.774	0.573
SAPS II	0.619	0.506	0.732	0.325	0.206	0.465	0.806	0.458	0.435	0.806	0.337

RF, random forest; LR, logistic regression; KNN, k-nearest neighbors; XGBoost, Extreme Gradient Boosting; SVM, support vector machine; DT, decision tree; SOFA, sequential organ failure assessment; SAPS II, simplified acute physiology score.

## Discussion

Sepsis persists as a prominent public health issue characterized by a poor prognosis, high mortality rates, and substantial utilization of healthcare resources, attributable to post-sepsis complications (33). The pathophysiology of liver injury in sepsis encompasses a range of interconnected mechanisms, including inflammatory overactivation, oxidative stress, mitochondrial and microcirculatory dysfunction, bacterial translocation, and adverse therapeutic effects (3, 34, 35). Although the liver possesses considerable defensive capacity, preventing it from being the most frequently involved organ in sepsis, once dysfunction occurs, the outcome is severe, with mortality rates reported ranging from 54 to 68%, exceeding that associated with failure of other organ dysfunctions or failures (36). Our study found that 3.2% of septic patients were diagnosed with SRLI within the first 24 h of ICU admission, a proportion that increased to 8.3% by the time of discharge. SRLI has been identified as an independent risk factor for mortality within 90 days among septic patients (37). Hence, early mitigation of liver injury is imperative for enhancing their prognosis (38). Nevertheless, achieving early detection and intervention remains challenging due to a range of obstacles.

In this retrospective study, we developed and externally validated ML models to predict the risk of SRLI in critically ill patients based on the MIMIC database. To effectively leverage high-frequency clinical measurements, often recorded multiple times daily, we incorporated both the maximum and minimum values of each variable as independent features. This strategy enabled the model to capture disease severity more accurately and to retain critical abnormal information, thus reducing potential bias arising from irregular sampling or measurement noise. Among all models, RF model significantly outperformed both conventional scoring systems (SOFA, SAPS II) and other ML algorithms, supported by DCA and robust performance metrics in both internal and external validation. Interpretability analysis via SHAP identified a predictive signature centered on hepatic and coagulation function. Our analysis quantified its impact, identifying specific threshold of the most important predictive features that may serve as data-driven benchmarks for clinical vigilance.

Serum total bilirubin was the most influential predictor, a routinely used biomarker for evaluating liver injury, owing to its low cost, technical simplicity, and reliable reproducibility. Elevated levels of total bilirubin indicate liver damage and deterioration of liver function (39). Thus, serum total bilirubin is used to diagnose SRLI as a crucial liver function index. This is consistent with our study, as patients with elevated total bilirubin levels are highly susceptible to developing SRLI. The SOFA score, frequently applied to evaluate multiorgan dysfunction and overall morbidity in critical illnesses, was also an important predictor in our study (40, 41). Previous studies have demonstrated its utility in predicting sepsis risk following liver transplantation (42). However, our study found that SAPS II and SOFA score showed suboptimal predictive performance compared to the ML models, suggesting that these conventional scoring systems may be inadequate for predicting SRLII. This is not entirely unexpected, since SOFA score is a composite evaluation tool encompassing six organ systems, including respiratory, renal, cardiovascular, hematologic, and hepatic parameters. Similarly, SAPS II primarily evaluates disease severity in ICU patients based on physiological variables and chronic health conditions (43). Hence, due to their multisystemic evaluation framework and reliance solely on serum bilirubin levels, both SOFA and SAPS II score fail to capture the multifaceted pathophysiological processes underlying SRLI, such as cholestasis, hepatocellular necrosis, or hepatic synthetic dysfunction. Notably, although SOFA alone may be insufficient for predicting SRLI, it contributed meaningfully to our ML model when integrated with other variables.

Other top contributors included INR and PT, highlighting the integral link between coagulopathy and hepatic synthetic dysfunction in sepsis. Notably, PT emerged as a highly influential predictor in our model. PT is a sensitive marker of hepatic synthetic function, with prolonged values indicating reduced production of coagulation factors during hepatocyte injury. Previous research has linked elevated PT levels to an increased risk of fulminant hepatic injury (44), highlighting the prognostic value in hepatic dysfunction. Although PT is not currently included in the diagnostic criteria for SRLI, its strong predictive performance in our analysis suggests that routine monitoring of PT in septic patients could serve as an early warning signal for impending liver injury. The LODS score is used to assess the severity of organ dysfunction in critically ill patients and is closely linked to the development of multiorgan dysfunction (45). LODS scoring, more sophisticated than SOFA score, assigns varying weights to different organ dysfunctions, with hepatic dysfunction receiving a relatively lower weight. Nevertheless, LODS plays an essential role in our ML model for predicting SRLI, possibly due to its incorporation of PT as one of the core variables for evaluating liver function. Overall, the laboratory parameters and scoring systems identified in our model are routinely available in clinical practice, which further enhances the practicality and applicability of ML-based prediction tools.

The RF algorithm is renowned for its capacity to integrate numerous covariates with minimal parameter tuning by aggregating multiple decision trees (46). This ensemble approach effectively reduces the risk of overfitting commonly seen in single-tree models. In this study, the RF model showed excellent performance compared to alternative models (LR, KNN, SVM, decision tree, XGBoost, and naïve Bayes) and clearly outperformed traditional scoring systems. These findings align with prior research. Hu et al. reported that the RF model exhibited superior prediction performance to other ML models in predicting mortality among sepsis patients readmitted to the ICU (47). Similarly, Devon Chang et al. confirmed that RF model exhibited superior accuracy in identifying liver fibrosis and cirrhosis (24).

Meanwhile, we employed SHAP interpreter technique to enhance the interpretability of RF model by assessing the impact of each variable on model output through SHAP values. This transparency enables clinicians to understand why the model assigns a higher risk score to a particular patient, thereby improving confidence in AI-assisted decision-making. Importantly, all variables included in our model are part of standard ICU monitoring process, ensuring that the tool remains practical and cost-efficient for clinical implementation. Another strength of our study lies in the external validation performed using an independent dataset from Nanjing Jinling Hospital, which helped mitigate overfitting and assess generalizability. Ultimately, the RF model consistently outperformed the other seven ML models in the external validation cohort, demonstrating robust predictive accuracy. Timely prevention and treatment of sepsis associated complications are crucial, as delayed diagnosis often precludes patients from receiving timely, higher-level interventions, potentially leading to serious adverse outcomes. Currently, no reliable diagnostic or screening method is available to identify patients at risk of developing SRLI. Therefore, our study addresses an important gap by offering a feasible and accurate prediction approach for early SRLI identification.

Nevertheless, our study had the following limitations and shortcomings. Firstly, the training cohort was sourced from MIMIC-IV database with a predominance of White person, which is distinct from external validation cohort. This discrepancy may have impacted the model performance. Secondly, the external validation was a monocentric retrospective cohort with a small sample size (*n* = 120), among which 9 patients (7.5%) experienced SRLI. This limited number of events might have introduced potential bias and affected the robustness of external validation results. We need more prospective cohort studies with substantial sample size to confirm the validity of our model. Thirdly, despite we used the MI method to address missing data, it was inevitable to suffer from bias. Finally, our ML models were based on data extracted from public database, some indicators correlated with liver function had more missing values exceeding 20%. Therefore, we had to abandon some of these indices. In addition, it should be noted that our definition of SRLI, which combines total bilirubin > 2 mg/dL (34.2μmol/L) with INR > 1.5, while specific for excluding non-hepatic hyperbilirubinemia, may lack sensitivity for detecting mild or early hepatic dysfunction, potentially reducing the sensitivity of our predictive model in detecting early SRLI. Despite these limitations, our model demonstrates robust and generalizable performance for predicting clinically significant SRLI.

## Conclusion

In conclusion, the machine learning models developed in this study demonstrated robust performance in predicting SRLI among patients with sepsis, with the RF model exhibiting the strongest predictive capability. These findings underscore the potential of ML to assist clinicians in early risk stratification and to support more timely and individualized management strategies. Nevertheless, prospective multicenter studies are warranted to further validate and refine the clinical utility of the proposed model in real-world settings.

## Data Availability

The original contributions presented in this study are included in this article/Supplementary material, further inquiries can be directed to the corresponding author.

## Supplementary material

Supplementary Figure 1 Assessment of distributional consistency between observed and imputed clinical variables using density plots.

Supplementary Figure 2 Calibration curves of the Random Forest model before and after isotonic regression.

Supplementary Figure 3 SHAP dependence plots for the ten most influential features.
