# Supplementary figures and images for: Early risk stratification of sepsis-related liver injury via machine learning: a multicohort study
Source: Front Med (Lausanne). 2026 Jan 27;13:1649101. doi: 10.3389/fmed.2026.1649101 (PMC12886363; doi:10.3389/fmed.2026.1649101)

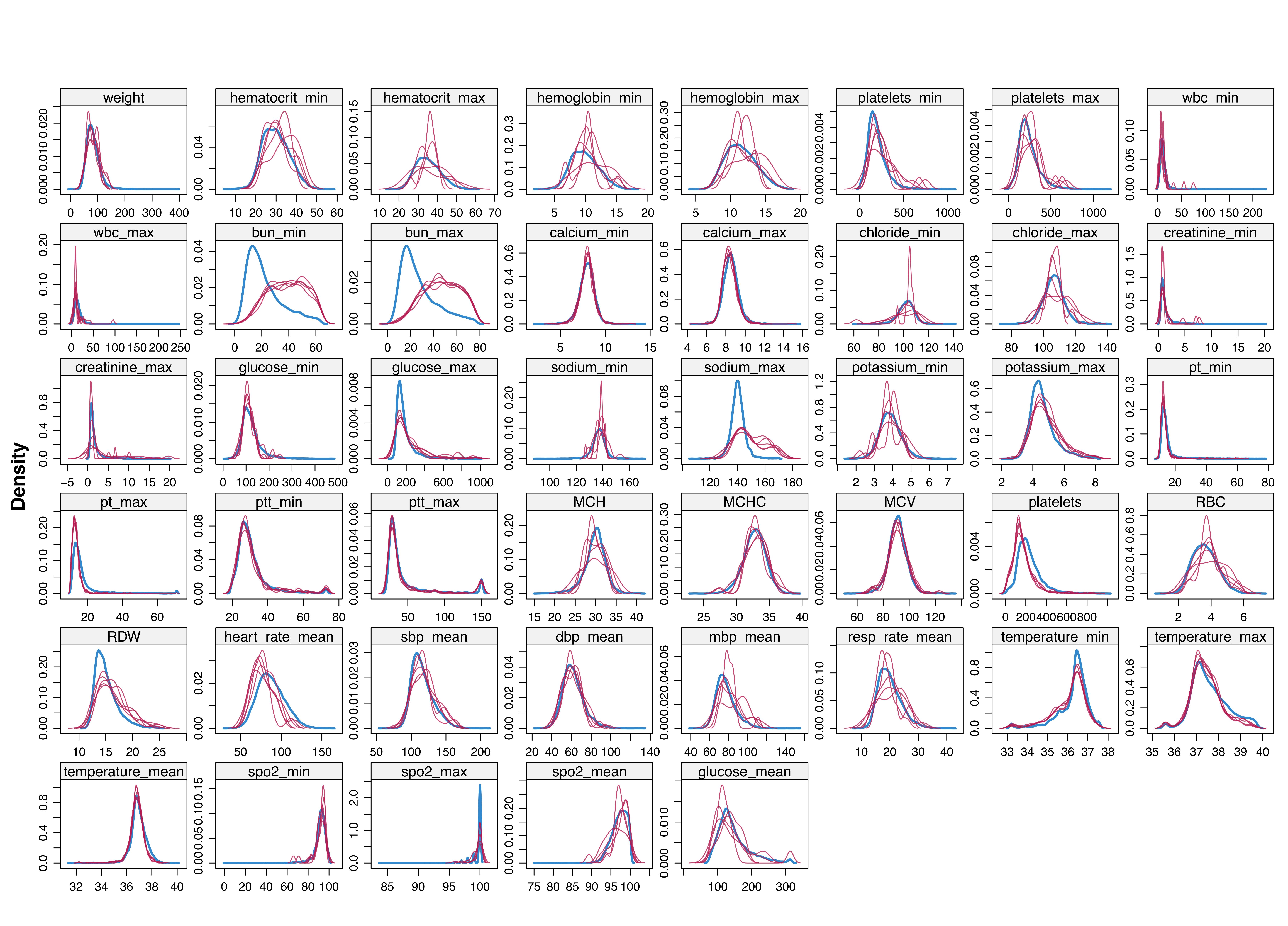

Supplement: Supplementary Figure 1 — Assessment of distributional consistency between observed and imputed clinical variables using density plots. [file Image_1.jpeg]

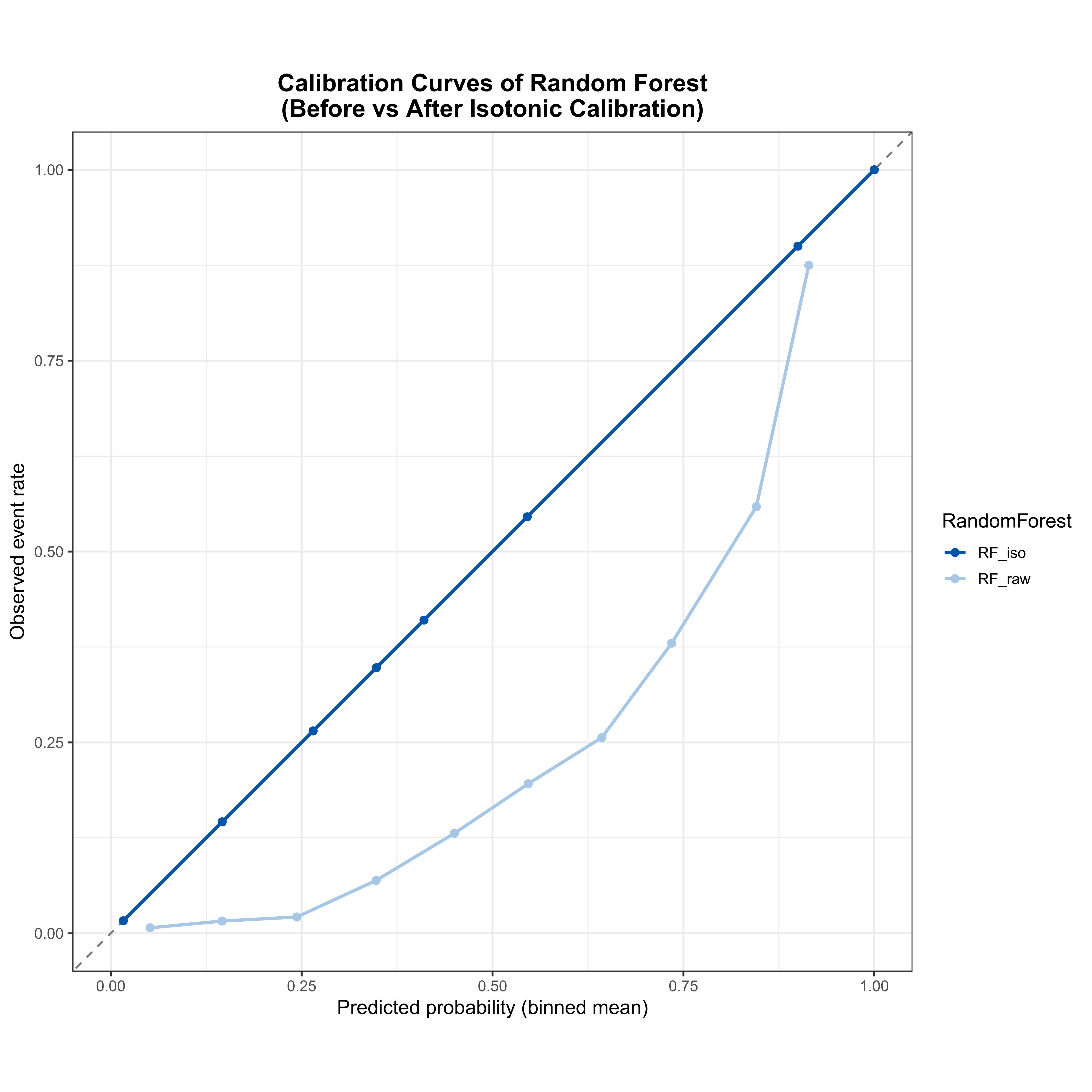

Supplement: Supplementary Figure 2 — Calibration curves of the Random Forest model before and after isotonic regression. [file Image_2.jpeg]

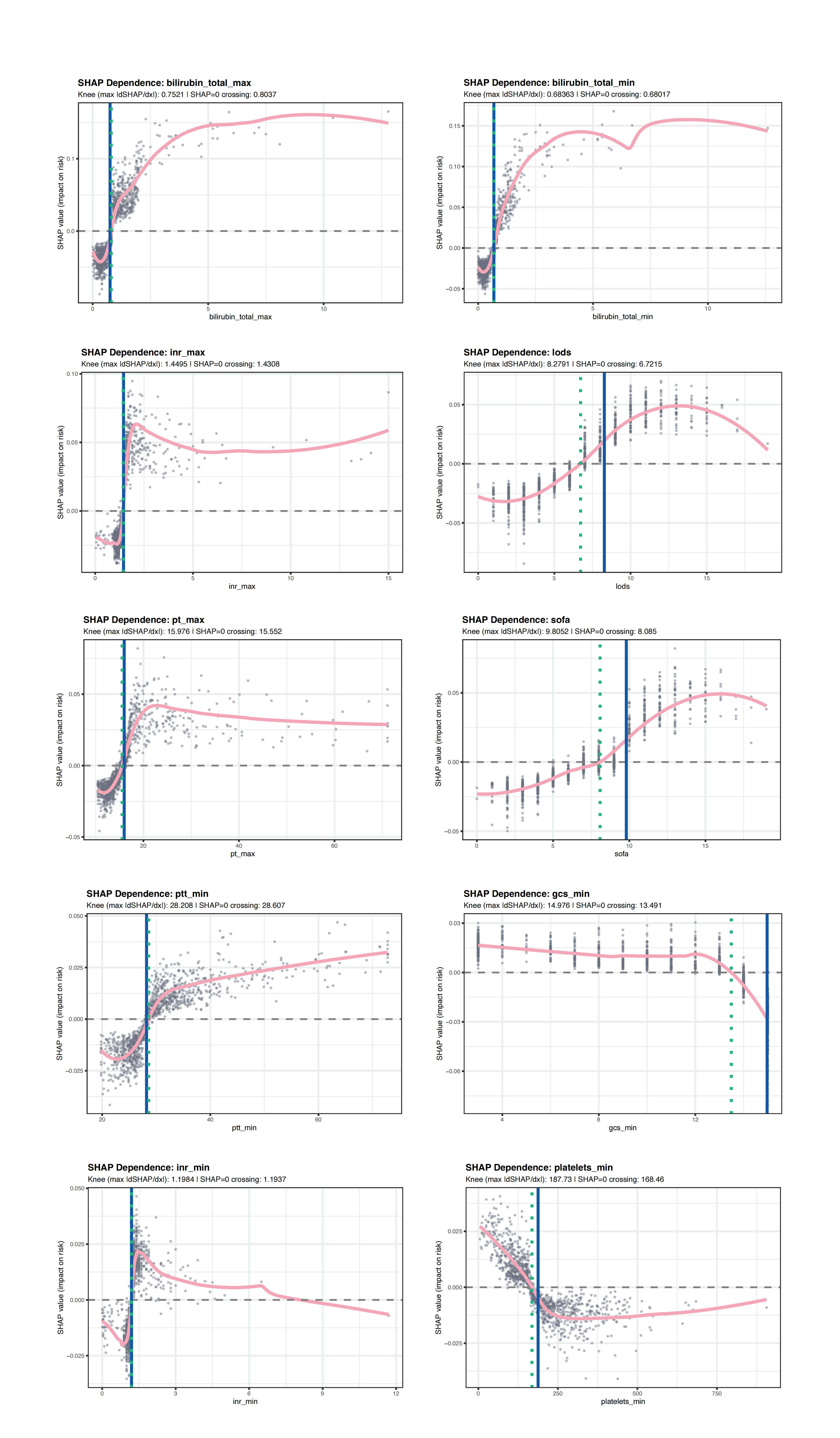

Supplement: Supplementary Figure 3 — SHAP dependence plots for the ten most influential features. [file Image_3.jpeg]
